# Supplementary material for: Cultivating capacities in community-based researchers in low-resource settings: Lessons from a participatory study on violence and mental health in Sri Lanka
Source: PLOS Glob Public Health. 2022 Nov 2;2(11):e0000899. doi: 10.1371/journal.pgph.0000899 (PMC10021324; doi:10.1371/journal.pgph.0000899)
Supplement: S1 Table — (DOCX) [file pgph.0000899.s001.docx]

**S1 Table.** Theme 1 evolution of early coding to final analytical framework

| **Illustrative preliminary coding for theme 1** | | | | **Final stage analytical framework for theme 1** | | |
| --- | --- | --- | --- | --- | --- | --- |
| **Extract from journal entry** | **First level codes** | **Second level codes** | **Parent code** | **Basic Theme** | **Organising Theme** | **Global Theme** |
| "Keeping their attention to the interview as they are not experienced in doing this, building trust and familiarity with the respondent." (ID02) | Interpersonal skills | Skill development | Learning during PEER project | Interpersonal skills | Skill and capacity development | Learning about research, violence and mental health |
| "I felt like my rephrasing of questions had improved as I had to ask questions in three different ways from the three participants (not the standard word to word questions in the interview guide) so that it would make sense to them, and I received very similar answers which makes me think that I got the questions through to them." (ID06) | Interviewing skills |  | Learning during PEER project | Interviewing skills |  |  |
| "I also learnt to observe my participants better as two of the three interviews happened through video chat. This enabled me to observe their body language and expressions thus, giving more meaning to their information." (ID06) | Observation skills |  | Learning during PEER project | Observation skills |  |  |
| "In this round, I developed skills to interview over the phone" (ID09) | Technology skills |  | Learning during PEER project | Technology skills |  |  |
| "In order to manage time, only the most important questions/facts were discussed in the interview. Also, a question that could be avoided because it had been answered before, was avoided" (ID08) | Time management + Organisational and documentation skills |  | Learning during PEER project | Time management and documentation skills |  |  |
| "Story stem helped to get much of information. Story stem was a new and interesting exercise for both the participants and me." (ID09) | Method knowledge |  | Learning during PEER project | Methods knowledge |  |  |
| "I would like to improve my note taking skills to gather information from lengthy interviews. This interview schedule on average spanned for around 2 hours, and I struggled a little to keep up with the speed of the interviewee as time passed due to fatigue and hand cramping. While round 3 interviews will not be as lengthy as round 2 interviews, if the core team can share some of effective note taking strategies that they use when conducting lengthy interviews, it would be beneficial to finding my own note taking styles." (ID05) | Note taking skills Fatigue Strategies | Learning gaps | Learning during PEER project | Identified capacity gaps |  |  |
| “I went into the first round of interviews, with expectations of how my peers/participants would answer to certain question. For example, I expected some of my participants to identify their religious/ethnic cohort as their community and I expected society’s majority conservative attitudes towards intimate relationships to heavily shape young people’s attitude towards intimate relationships – especially premarital relationships...The interviews did not unravel the way that I expected it to. All my participants had a very broad, inclusive, and sometimes an intellectual/ value-based definition of who their community is – none bound by identifiers such as religion and ethnicity. Secondly, through my participants, I was able to understand that young people have an openness and flexibility towards intimate relationships, and it is markedly different to attitudes held by previous generations. Overall, the round 1 interviews helped me understand attitudes and thoughts of a broader network of young people, through accounts gathered through my chosen peers/friends for this study. And it made me realize that my expectation of young people was markedly different to reality.” (ID05) | Expectations  Understanding | Learning about myself + Learning subject knowledge + Learning about study participant experience | Learning during PEER project | Shifts in perspective and assumptions | Empathy and personal growth |  |
| "It was a great experience for me. This hard time teaches me and shown me my capacity [sic]. I actually feel good” (ID20). | Capacity  Self-awareness | Learning about myself | Learning during PEER project | Skill application as empowering |  |  |
| “This made me feel passionate about my career in this field of social work because then I could help these young women” (ID21) | Passion  Motivation | Learning subject knowledge + Learning about myself | Learning during PEER project | Increased passion/motivation for working in certain fields and/or select issues |  |  |
| "And learned some different aspects and angles on parent – young adult relationships. And this research will help me personally when I become a parent". (ID11) | Personal application | Learning about myself | Learning during PEER project | Application to personal lives and lived experience |  |  |
| "Other than that, many sensitive matters were discussed. While on that subject I myself felt disgusted at the society and the participant’s mother reacted to the examples(stories) poorly. I felt that it was a hindrance" (ID17) | Emotions Disgust | Learning about myself + Interpersonal challenges | Learning during PEER project + (Nature of) Role or Project Challenges | Learning induced emotions |  |  |
| "During this round I got a glimpse of the considerations that must be factored in when delivering a support service or a care package for victim survivors of violence. As my participants and I engaged in a discussion about the existing support available, most of them were not affordable, accessible, and unable to handle incoming traffic. While having this conversation I realized that one size does not fit all, hence how do you ensure that the support or supports that are made available is helpful to as many victim-survivors of violence as possible is a question that I reflected on. This may entail considering the geographical location, vulnerabilities to accessibility, preferences of young victim-survivors of violence etc. Another key consideration would be training key personnel – including healthcare providers to inculcate ethics, values and practices to ensure responsible and accountable service delivery, to destigmatize service provider attitudes towards mental health, sexual and reproductive health services and accessing support services." (ID05) | Delivering support services Design considerations Access Learning Reflection | Learning for intervention development + Learning subject knowledge + Learning about myself + Reflections about Sri Lanka and its issues | Learning during PEER project | Application of intellectual and emotional learning towards identifying community solutions and problem solving | Implications of researcher learning for future community development |  |
| "I observed that they were curious about the things they did not know. I understood that it is important to have the knowledge, contacts and training to guide young women from the grassroot level to places that can help them. I believe if I receive the necessary training, I can be a volunteer to spread awareness to these young women in the communities." (ID21) | Curiosity Observation Personal application | Learning about myself + Learning for intervention development + Learning subject knowledge + Reflections about Sri Lanka and its issues | Learning during PEER project | CBRs as changemakers for strengthening other community members’ capacities |  |  |
