# Supplementary material for: Cultivating capacities in community-based researchers in low-resource settings: Lessons from a participatory study on violence and mental health in Sri Lanka
Source: PLOS Glob Public Health. 2022 Nov 2;2(11):e0000899. doi: 10.1371/journal.pgph.0000899 (PMC10021324; doi:10.1371/journal.pgph.0000899)
Supplement: S2 Table — (DOCX) [file pgph.0000899.s002.docx]

**S2 Table. Thematic findings: Global, organising, and basic**

| **Global Theme 1 – Learning about research, violence and mental health:** All reflections on what was learned or not across the parent project, including but not limited to skills, knowledge, personal growth | | |
| --- | --- | --- |
| **Organising Themes** | **Description** | **Basic Themes** |
| Skill and capacity development | Reflections on skills developed, strengthened, or sought in the project lifetime; development of subject and methodological knowledge | 1. Interpersonal skills 2. Interviewing skills 3. Observation skills 4. Technology skills 5. Time management and documentation skills 6. Methods knowledge 7. Identified capacity gaps |
| Empathy and personal growth | Content capturing CBR learning about themselves – their ideas, perceptions, beliefs, values; having their own position on issues challenged or further developed. This includes reflections on noticing and connecting with participants’ emotional and experiential accounts, and personal emotional experiences connected to a learning moment. | 1. Shifts in perspective and assumptions 2. Skill application as empowering 3. Increased passion/motivation for working in certain fields and/or select issues 4. Application to personal lives and lived experience 5. Learning induced emotions |
| Implications of researcher learning for future community development | Reflections on possible intervention development; views on state of study topics in the local context; and implications for participating in future efforts to address perceived needed change | 1. Application of intellectual and emotional learning towards identifying community solutions and problem solving 2. CBRs as changemakers for strengthening other community members’ capacities |
| **Global Theme 2 – Navigating personal-professional boundaries:** Data related to the CBRs’ relationships with participants and what it was like to navigate a dual role with (known) peers | | |
| **Organising Themes** | **Description** | **Basic Themes** |
| Pros of familiarity | Advantages or positives of knowing participants pre-project | 1. Perceived improved quality of conversations and information 2. Enhanced comfort with sensitive material 3. Willingness to commit and engage in interviews without (monetary) incentives |
| Cons of familiarity | Disadvantages or difficulties related to knowing participants prior to parent study | 1. Difficulty establishing boundaries 2. Impacts on disclosure, e.g., under- or over-sharing due to common peers 3. Distraction and conversational diversion |
| Strategies for navigating boundaries | Actions or choices taken by CBRs to navigate perceived healthy and professional CBR-participant boundaries, often to support CBR and/or participant wellbeing and data quality | 1. Expectation and disclosure management 2. Reinforcing boundaries during interview process 3. Considerations for participant selection 4. Selecting mode and setting of interviews to aid quality/connection, plus COVID’s impact on choice |
| **Global Theme 3 – Challenges in sensitive participatory research:** All data reflecting on difficulties experienced across the project cycle, their impacts, and management | | |
| **Organising Themes** | **Description** | **Basic Themes** |
| Interpersonal challenges | Challenges between CBRs and other people impacting achievement of project goals | 1. Maintaining boundaries 2. Participant availability 3. Managing bystanders 4. Facing distractions 5. CBR and participant emotions 6. COVID-19 impact on interpersonal challenges |
| Time management challenges | Challenges related to delays, ability to meet deadlines and goals | 1. Difficulty managing time during interviews 2. Difficulty organising appointments 3. COVID-19 impacts on timelines |
| Health and safety challenges | All data regarding individual or communal health, safety and wellbeing creating difficulties | 1. CBRs’ personal health 2. COVID-19 impacts on health and safety of all parties |
| Technology challenges | Any difficulties created or worsened by technology access, skills, stability | 1. Internet connectivity 2. Role of weather 3. COVID-19’s role in technology challenges |
| Strategies for navigating challenges | Choices or actions taken to try and overcome or face challenges in the project; how well they worked; who was involved, etc. | 1. Communication strategies 2. Planning strategies 3. Technology strategies |
| **Global Theme 4 – Experiences of (support from) the core team**: All data reflecting on the role of and experiences with the core research team. | | |
| **Organising Themes** | **Description** | **Basic Themes** |
| Conditions of working with the core team | Perceptions of the core team’s role and ability to meet CBR needs appropriately. Extent to which CBRs were able and enabled to identify and vocalise needs throughout project lifecycle. Circumstances influencing CBRs’ interpersonal dynamics with core team. | 1. CBRs valued (support from) core team 2. CBRs felt able to seek assistance 3. Core team accessibility and presence; lack of isolation concerns 4. Flexibility 5. Anticipation of needs (limiting reactionary support) |
| Provision: Comprehensive communication | Role of communication with core team for CBR experience | 1. Multi-method communication modes available 2. Communication in all languages, language-matched support |
| Provision: Role of interactive forums | Reflections on the role of capacity strengthening activities including pre- and in-service training and inter-round debriefs | 1. Increased CBR confidence 2. Value of training programme, including roleplay, and sharing concerns pre-fieldwork 3. Supportive role of supplementary practice sessions 4. Debriefs providing opportunity to reflect on limitations and continue sharing |
| Provision: Consumable resources | Reflections on material inputs and their impact on CBRs, participants and project outcomes | 1. Project allowances 2. Further support brochures 3. Vouchers and other gestures 4. IT equipment |
