# Supplementary material for: Cultivating capacities in community-based researchers in low-resource settings: Lessons from a participatory study on violence and mental health in Sri Lanka
Source: PLOS Glob Public Health. 2022 Nov 2;2(11):e0000899. doi: 10.1371/journal.pgph.0000899 (PMC10021324; doi:10.1371/journal.pgph.0000899)
Supplement: S3 Table — (DOCX) [file pgph.0000899.s003.docx]

**S3 Table. Expanded recommendations for supportive team dynamics in sensitive participatory research in low resource settings**

| **Priority Area** |
| --- |
| ***Team Communication Culture* (C1-C7)** |
| 1. Explicitly convey messages of openness to and appreciation for discussion including alternative and/or dissenting views with community-based and junior colleagues from project inception, and reinforce such messages throughout the project cycle to minimise self-silencing [2]. |
| 1. Agree clear roles and responsibilities in the team that reflect accountability from all sides regardless of seniority and/or experience. Consider providing handbooks delineating roles and responsibilities (and standards as per below) and refer to them as necessary throughout the project cycle. |
| 1. Agree standards of comportment for all including zero-tolerance policies where necessary and considering the sensitivity of research subjects. Clear expectation management from all sides fosters professionalism regardless of role. Explicate non-negotiable standards and co-agree others in training and provide them in multiple formats, e.g. a role handbook, to all team members. |
| 1. Consider pairing CBRs with a core team member proficient in CBRs’ first language, both of whom will then remain stable and accessible, communicating availability for supervision and discussions throughout the project.* |
| 1. Confirm team communication mechanisms, whether and under what circumstances particular mechanisms should be selected (e.g. debriefs vs. team meetings), and offer multiple methods for connection (e.g. phone, email, messaging app, in-person, group and one-to-one discussions, etc.). The latter is particularly important for settings with unreliable or deficient access to telecommunication resources and/or CBRs who may not typically have access to all communication devices. |
| 1. Core team members to maintain availability – reliably – through multiple channels during the project. |
| 1. Normalise respectful, consistent, and responsive feedback practices amongst all colleagues*, e.g. through group debriefs, role play, and modelling openness to feedback in view of junior colleagues. |
| ***Project Design* (D1-D7)** |
| 1. Structure data collection phases to allow for repeated opportunities for CBRs to apply, test, and improve upon data collection skills, e.g. building in multiple, piloting and/or mock rounds, as one-off data collection events limit learning and growth. |
| 1. Build-in intentional and protected opportunities for self-* and team-based reflection with CBRs, e.g. paired debriefs with a core team member, inter-round workshops. Consider synthesising such reflections in a post-project review. |
| 1. Anticipate the need for and be supportive of additional practice sessions for CBRs.* We first held a two-day training for the study (in addition to previous parent project training), but reconvened for additional time after identifying outstanding needs and anxieties. CBRs took initiative to self-organise practice sessions, and core team members set up extra one-on-one mock sessions. |
| 1. Support decision-making by CBRs by creating ‘choice opportunities’ throughout the project lifecycle. Even with set parameters for manageability and standards across the team, power in some key decisions could favour CBRs. Our CBRs chose topics by geographic and gendered teams in the parent study; a choice which benefitted the evidence base and team development as novel topics were selected, complementing original team priorities. |
| 1. Embrace, model and expect flexibility when it will benefit team wellbeing and/or evidence, e.g. granting additional time or support.* |
| 1. Design mechanisms for preventing and managing adverse events; e.g. a project-appropriate incident management system for diverse fieldwork challenges (e.g. vehicle breakdowns, project material loss/theft, participant distress). These mechanisms may never be activated but creating them in the moment risks mishandling responses. |
| 1. Plan sufficient time and space for CBRs to discuss, identify and/or lead dissemination activities. If desired and safe, this may involve taking active steps to visibilise CBRs within their preferred circles, positioning them as knowledgeable change agents for future efforts within their communities [2]. |
| ***Foundational Training(s)* (T1-T7)** |
| 1. To support CBR’s emotional safety, particularly in violence, mental health and otherwise sensitive research [7], introduce evidence-informed strategies for managing discomfort, practicing reflexivity (e.g. research journals, debriefs), and preparing for personal beliefs and/or assumptions to be challenged or even changed.* |
| 1. Create interactive, multi-method opportunities to practice navigating diverse interpersonal dynamics, e.g. role play and mock data collection scenarios, particularly those anticipating potential challenges of managing bystanders, interruptions, distress, and de-escalation.* Booster practice may be needed. |
| 1. Develop multiple opportunities in training to practice and receive feedback on data collection skills, such as active listening, rewording, simplifying and assessing question suitability, and when/how best to probe in interview-based studies. This is critical in sensitive research as data collection moments may be re- or vicariously-traumatising to participants or CBRs respectively [7]. |
| 1. Thoroughly discuss and consider co-developing ‘further support’ materials *with* CBRs to build their confidence should study participants require their assistance to access e.g. violence and/or mental health support services. Furnishing CBRs with pre-made materials without in-depth discussion may leave CBRs anxious and/or under-prepared to respond in situ. These materials are also applicable to CBRs themselves as part of self-care. |
| 1. Reenforce messages that while flexibility is necessary in projects, CBRs should not (be expected to) compromise their own safety and wellbeing to accommodate participants or other project needs [7].* |
| 1. Dedicate time for practical and generalised professional skills including documentation practices for the project (e.g. note taking); digital resource, time and expectation management. |
| 1. Foster confidence in using all technologies and tools in the project, including for data management. Do not presume all parties possess the requisite items/devices nor are competent and comfortable (independently) applying them – practice how they should be used within the project itself*. Trial technology trouble-shooting strategies to support confidence in data collection*. Our CBRs selected to record fieldnotes in whichever form they felt most confident to produce high quality data, accommodating different technological fluencies (digital or handwritten forms). |
| ***Human and Consumable Resources* (R1-R9)** |
| 1. Dedicate strategic time and effort to identify, recruit and retain suitable CBRs; more time on ‘smart’ hiring may reduce problematic team dynamics and/or lower team attrition later – benefitting team and evidence quality. Consider using trainings as a two-way job interview for both CBRs and core team members to assess fit and introduce a mutual trial period when possible. Make efforts, where possible, to recruit those motivated by research relevance to their personal and communities’ lives, which may support inclusion of those most affected and most likely to benefit from research efforts [50]. |
| 1. Consider exercises to identify strong team leaders amongst CBRs early in the project who may support motivating and learning efforts amongst CBRs differently to the core team. Rigorous, dynamic training may provide multiple scenarios to identify leadership capacities in CBR (candidates). |
| 1. Build-in extra time to allow for project slippage due to unforeseen challenges.* |
| 1. Ensure necessary and multiple technologies are available for all parties, giving alternatives and/or catch-up time should primary technologies malfunction. This is especially salient for remote data collection methods. |
| 1. Ensure project tools are provided in multiple formats (e.g. digital and print; cloud and email) and are accessible (e.g. consider language and literacy proficiencies and (dis)ability). |
| 1. Provide reasonable allowances for all parties participating in cost-incurring activities, e.g. internet, travel, phone, food, supply credit for CBRs *and* participants. Some items may be deemed budget ‘comforts’ by decision-makers, but in practice, in sensitive studies, are more often viewed as wellbeing essentials for CBRs and participants alike. Seek ways of reconciling budget allocation differences where possible for such investments. |
| 1. If appropriate, consider and co-agree end of project gifts of thanks and compensation for participants *with* CBRs (e.g. letters of appreciation, vouchers for participants; this was appreciated in our own project due to challenging pandemic conditions which permitted participants to meet diverse needs without prescription). |
| 1. As appropriate, consider offering gestures recognising CBRs during and post-service, e.g. letters of service documenting strengths for future employers, performance-based bonuses, writing references for new roles or higher education opportunities. |
| 1. Offer wellbeing resources for all parties – particularly in violence and mental health projects, e.g. further support leaflets for participants, external clinical supervision on demand or other appropriate mental health support for CBRs [7]; simple team away days; and core team time for discussing challenges with CBRs. |
| ***Considerations for Volatile Research Conditions^†^* (V1-V9)** |
| In addition to tackling sensitive research, teams may face additional and changeable working conditions due to natural or human-made disasters, (civil) unrest and conflict, health crises, and more. Drawing on our experience navigating the COVID-19 pandemic and a parallel economic crisis, we propose: |
| 1. Identifying potential challenging scenarios and project ‘threat’ levels with CBRs to anticipate (un)likely conditions.* |
| 1. Developing mitigation strategies with high, medium and low levels of control for CBRs to consider how they could adapt to changing conditions.* We established and trained on contingency plans for changing mobility restrictions during COVID-19 so all CBRs could modify data collection modes safely, confidently and automatically. |
| 1. Jointly discussing how CBRs would feel in each scenario, what and how best to deploy strategies* with appropriate inputs from the core team. |
| 1. Formalising and reviewing safety plans, including extraction planning. |
| 1. Ensuring appropriate insurance is in place for field-based team members. This may include health, accident and transportation insurance coverage. |
| 1. Providing project-tailored health and safety kits for fieldwork/travel. We expanded standard first aid kits with COVID-19 and environmentally relevant safety supplies. |
| 1. Collectively anticipating and accepting undesirable shifts in the project may be necessary including additional and/or different uses of time, training, resources, and project goals.* We, for example, extended CBR contracts twice during COVID-19, accommodating any additional employment commitments beyond our project. |
| 1. Openly discussing the potentiality for project interruption and/or cessation in extreme circumstances and reiterating that no research is more important than team or participant wellbeing.* |
| 1. Transparently agreeing employment conditions and commitments under such circumstances to foster mutual trust. The core team redesigned our project amidst COVID-19 to honour original offers of employment to CBRs, with both sides accepting conditions such as length of contract could not be fully re-matched. |
| * Denotes recommendations which could be (partially) independently enacted by CBRs  † While we stress these recommendations for volatile contexts, all items are relevant and reasonable for projects deemed low risk at their inception as field conditions can and do change swiftly. |
