# Supplementary material for: Cultivating capacities in community-based researchers in low-resource settings: Lessons from a participatory study on violence and mental health in Sri Lanka
Source: PLOS Glob Public Health. 2022 Nov 2;2(11):e0000899. doi: 10.1371/journal.pgph.0000899 (PMC10021324; doi:10.1371/journal.pgph.0000899)
Supplement: S1 File — (DOCX) [file pgph.0000899.s004.docx]

**S1. Alternative language abstracts**

**Sinhala abstract**

අඩු සහ මධ්‍යම ආදායම්ලාභී රටවල ප්‍රචණ්ඩත්වය සහ මානසික සෞඛ්‍යය පිළිබඳව අවශ්‍ය සාක්ෂි ජනනය උදෙසා ප්‍රජා-පාදක දත්ත රැස්කරන්නන් (Community-based data collectors) මත දැඩි ලෙස රඳා පවතින සහභාගීත්ව නිරික්ෂණ ක්‍රමවේද (Participatory methods) ප්‍රචලිතිව භාවිතා වන නමුත්, ප්‍රචණ්ඩත්වය සහ මානසික සෞඛ්‍යය ගැටළු වලට ප්‍රතිචාර දැක්වීමට ඇති සම්පත් ඉතාමත් සීමිත වේ. තවද, පර්යේෂණ සහභාගිවන්නන්ගේ යහපැවැත්ම කෙරෙහි වැඩි අවධානයක් යොමු වී ඇති අතර, වෘත්තීමය පර්යේෂකයන් සහ ප්‍රජා-පාදක පර්යේෂකයන්ගේ (Community-based researchers) භූමිකා-විශේෂිත අවශ්‍යතා කෙරෙහි අඩු අවධානයක් යොමුවි ඇත. මෙම සම-නිෂ්පාදිත පත්‍රිකාව අඩු සම්පත් සැකසුම් තුළ සංවේදී සහභාගීත්ව ව්‍යාපෘතිවල ප්‍රජා-පාදක පර්යේෂකයන්ගේ යහපැවැත්මට සහ සංවර්ධනයට දායක වීම සඳහා ප්‍රතිලාභ, අභියෝග, සහ නිර්දේශ හඳුනා ගැනීමට එක් සම-පර්යේෂකයන් කණ්ඩායමක (Peer researcher group) අත්දැකීම් පාදක කොටගෙන ඇත.

ශ්‍රී ලංකාවේ ‘තරුණයින්, ප්‍රචණ්ඩත්වය සහ මානසික සෞඛ්‍යය’ පිළිබඳ මිශ්‍ර ක්‍රම අධ්‍යයනයකට සහය දැක්වූ ප්‍රජා-පාදක පර්යේෂකයන් ලෙස හදුන්වන සම-පර්යේෂකයන් 21 දෙනෙකු දත්ත එකතු කිරීමේ වට තුනකින් පසුව, ප්‍රත්‍යාවර්තී ව්‍යුහගත සඟරා ඇතුළත් කිරීම් (Reflexive structured journal entries) 63 ක් ඉදිරිපත් කරන ලදී. මෙම තේමාත්මක විශ්ලේෂණයට ‘අට්‍රයඩ්-ස්ටාර්ලින්ගේ ක්‍රමවේදය’ (Attride-Stirling’s method) භාවිතා කිරීමෙන්: පර්යේෂණ පිළිබඳ, ප්‍රචණ්ඩත්වය සහ මානසික සෞඛ්‍යය පිළිබඳ, පුද්ගලික-වෘත්තීය සීමාවන් පිළිබඳ, සංවේදී පර්යේෂණවල අභියෝග පිළිබඳ, සහ මූලික කණ්ඩායමේ සහාය පිළිබඳ සම-පර්යේෂකයන්ගේ අත්දැකීම් ගවේෂණය කරන ලදි.

විවිධත්වයෙන් පොහොසත් ප්‍රජා-පාදක පර්යේෂකයන් පිරිසකගේ අත්දැකීම් විශ්ලේෂණය කරන ශ්‍රී ලංකාවේ පළමු අධ්‍යයනය මෙයයි. මෙම අධ්‍යයනයේ අරමුණ වන්නේ සම්පත් සීමා සහිත සහ වෙනස්වන සුලු සැකසුම් තුළ සංවේදී සහ චිත්තවේගීය වශයෙන් දුෂ්කර කාර්යයක් කිරීමට ප්‍රජා-පාදක පර්යේෂකයන් මත විශ්වාසය තබන ගෝලීය සෞඛ්‍ය සහ සංවර්ධන ක්‍රියාකාරීන් දැනුවත් කිරීමයි. මෙම සහභාගීත්ව පර්යේෂණය ප්‍රජා-පාදක සහ වෘත්තීය පර්යේෂකයන් අතර අන්‍යෝන්‍ය ඉගෙනීමේ අවස්ථාවක් ලෙස සලකා බැලීමෙන් ගෞරවනීය වූ කණ්ඩායම් ගතිකත්වයන් පෝෂණය කිරීමට සහ සියලු පාර්ශව සඳහා උත්පාදක සහ සුරක්ෂිත බැවින් යුත් සම-නිෂ්පාදන ව්‍යාපෘති නිර්මාණය කිරීමට පාදක වූ ප්‍රායෝගික පරතරයන් සහ අවස්ථා අපි හඳුනා ගනිමු. සන්නිවේදනය, පුහුණුව, මානව සහ පරිභෝජන සම්පත්, ව්‍යාපෘති සැලසුම් කිරීම සහ අස්ථායී පර්යේෂණ තත්වයන් තුල හසුරුවීම පිලිබදව ගත් අධ්‍යාශයික තිරණ කණ්ඩායම තුල විවිධ පුද්ගලික සහ වෘත්තීය හැකියාවන් ශක්තිමත් කරන ලදී. මේ නිසා කෙටිකාලීන හා දිගු කාලීන සාක්ෂිවල ගුණාත්මක භාවය ; ප්‍රචණ්ඩත්වය සහ මානසික සෞඛ්‍යය වැනි තීරණාත්මක ගැටළු සම්බන්ධයෙන් ඉහළ බැර - අඩු සම්පත් සන්දර්භයන් තුල ක්‍රියා කිරීම ආදී ක්‍රියාමාර්ගයන් පිලිබද තොරතුරු රැස් කිරීමට ඵලදායී විය.

**Tamil abstract**

சமூகம் சார்ந்த தரவு சேகரிப்பாளர்களை பெரிதும் நம்பியிருக்கும் பங்கேற்பு முறைகள், குறைந்த மற்றும் நடுத்தர வருமானம் கொண்ட நாடுகளில் வன்முறை மற்றும் மனநலம் குறித்த மிகவும் தேவையான ஆதாரங்களை வழங்க பிரபலமடைந்து வருகின்ற போதிலும் பதிலளிப்பதற்கு குறைவான ஆதாரங்களே உள்ளன. ஆராய்ச்சி பங்கேற்பாளர்களின் நல்வாழ்வில் அதிக கவனம் செலுத்தப்பட்ட போதிலும், அத்தகைய ஆய்வுகளில் தொழில்முறை ஆராய்ச்சியாளர்கள்; சமூகம் சார்ந்த ஆராய்ச்சியாளர்களின் பங்கு குறைவான கவனத்தைப் பெறுகின்றது. குறைந்த வள அமைப்புகளில் உணர்திறன் மிக்க பங்கேற்பு திட்டங்களில் சமூகம் சார்ந்த ஆராய்ச்சியாளர்களின் நல்வாழ்வு மற்றும் மேம்பாட்டை ஆதரிப்பதற்கான பரிந்துரைகள், தேவையான வெகுமதிகள், மற்றும் எதிர்கொள்ளும் சவால்களை அடையாளம் காண இந்த இணை-தயாரிக்கப்பட்ட கட்டுரை ஒரு குழுவின் அனுபவத்திலிருந்து தரவுகளைப் பெறுகிறது.

இலங்கையில் இளைஞர்கள், வன்முறை மற்றும் மனநலம் பற்றிய கலப்பு முறை ஆய்வுக்கு ஆதரவளிக்கும் 21 சமூகம் சார்ந்த ஆராய்ச்சியாளர்களின், மூன்று சுற்று தரவு சேகரிப்பைத் தொடர்ந்து, 63 பிரதிபலிப்பு கட்டமைக்கப்பட்ட கட்டுரை உள்ளீடுகளை சமர்ப்பித்தனர். வன்முறை மற்றும் மனநலம் பற்றி சக ஆராய்ச்சியாளர்களின் கற்றலை ஆராய, கருப்பொருள் பகுப்பாய்விற்கு Attride-Stirling இன் முறையைப் பயன்படுத்தினோம்; தனிப்பட்ட-தொழில்முறை எல்லைகள்; உணர்திறன் ஆராய்ச்சியில் சவால்கள்; மற்றும் முக்கிய மத்திய குழுவின் ஆதரவு.

இலங்கையின் பல்வேறு சமூக அடிப்படையிலான ஆய்வாளர்களின் அனுபவங்களைக் கைப்பற்றும் இலங்கையின் முதல் ஆய்வானது, வளங்கள் மட்டுப்படுத்தப்பட்ட மற்றும் நிலையற்ற அமைப்புகளில் உணர்திறன் மற்றும் உணர்ச்சி ரீதியாக கடினமான செயற்பாடுகளில் ஈடுபட இத்தகைய திறமைகளை நம்பியிருக்கும் அதிகரித்து வரும் உலகளாவிய சுகாதார மற்றும் அபிவிருத்தி காரணிகளை நோக்கமாகக் கொண்டுள்ளது. சமூகம் சார்ந்த மற்றும் தொழில்முறை ஆராய்ச்சியாளர்களிடையே பரஸ்பர கற்றலுக்கான வாய்ப்பாக இந்த பங்கேற்பு ஆராய்ச்சியைப் பார்க்கிறோம், அனைத்துத் தரப்பினருக்கும் இடையே மரியாதைக்குரிய குழு இயக்கவியலை வளர்ப்பதற்கும், மற்றும் பாதுகாப்பான கூட்டுத் தயாரிப்புத் திட்டங்களை உருவாக்குவதற்கும், இடையே உள்ள பயிற்சி இடைவெளிகளையும் அதற்கான வாய்ப்புகளையும் நாங்கள் அடையாளம் காண்கிறோம். தகவல்தொடர்பு, பயிற்சி, மனித மற்றும் நுகர்வு வளங்கள், திட்ட வடிவமைப்பு மற்றும் நிலையற்ற ஆராய்ச்சி நிலைமைகளுக்கு வழிசெலுத்துதல் ஆகியவை பல தனிப்பட்ட மற்றும் குழுக்கள் முழுவதுக்குமான தொழில்முறை திறன்களை வலுப்படுத்தலாம், குறுகிய மற்றும் நீண்ட கால தரமான சான்றுகள் மற்றும் வன்முறை உட்பட முக்கியமான சிக்கல்களில்; மன ஆரோக்கியம், அதிக சுமை, குறைந்த வள சூழல்களை அறிய உதவும்.
