# Supplementary material for: Cultivating capacities in community-based researchers in low-resource settings: Lessons from a participatory study on violence and mental health in Sri Lanka
Source: PLOS Glob Public Health. 2022 Nov 2;2(11):e0000899. doi: 10.1371/journal.pgph.0000899 (PMC10021324; doi:10.1371/journal.pgph.0000899)
Supplement: Attachment — Submitted filename: COMMENTS TABLE_Responses to review_8.7.22.pdf [file pgph.0000899.s006.pdf]

PGPH-D-22-00472: Cultivating capacities in community-based researchers in low-resource settings: Lessons from a participatory study on violence and mental health in Sri Lanka - *PLOS Global Public Health*

Reviewer comment	Author reply	Location of changes
**Editor comments**		
General comments		
Please include a complete copy of PLOS' questionnaire on inclusivity in global research in your revised manuscript. Our policy for research in this area aims to improve transparency in the reporting of research performed outside of researchers' own country or community. The policy applies to researchers who have travelled to a different country to conduct research, research with Indigenous populations or their lands, and research on cultural artefacts. The questionnaire can also be requested at the journal's discretion for any other submissions, even if these conditions are not met. Please find more information on the policy and a link to download a blank copy of the questionnaire here: https://journals.plos.org/globalpublichealth/s/best-practices-in-research-reporting. Please upload a completed version of your questionnaire as Supporting Information when you resubmit your manuscript.	The PLOS Inclusivity in global research questionnaire has been completed and is now a supplementary document as per Editor request.<br><br>A sentence was also added, as per the Questionnaire's suggestion, to our Ethics subsection signposting to Supplement 3.	Ethics lines 265-266<br><br>Supplement 3 (S3)

In the online submission form, you indicated that "Our dataset is not open access to ensure anonymity of socially-connected study participants who may be re-identified through this study's data and authorship." All PLOS journals now require all data underlying the findings described in their manuscript to be freely available to other researchers, either 1. In a public repository, 2. Within the manuscript itself, or 3. Uploaded as supplementary information.<br>This policy applies to all data except where public deposition would breach compliance with the protocol approved by your research ethics board. If your data cannot be made publicly available for ethical or legal reasons (e.g., public availability would compromise patient privacy), please explain your reasons by return email and your exemption request will be escalated to the editor for approval. Your exemption request will be handled independently and will not hold up the peer review process, but will need to be resolved should your manuscript be accepted for publication. One of the Editorial team will then be in touch if there are any issues.	We recognise the value of providing raw data where possible and considered carefully where to seek publication understanding the PLOS GPH priority for data availability.<br><br>On November 21, 2021, we inquired with a member of the PLOS GPH editorial board (via email) prior to submission to understand whether an exception in this case would be considered. We explained that we felt this was the right forum for this specific piece, but also recognised the challenge of not having a fully open dataset.<br><br>Our authors are very closely linked with participants, including family, friends, neighbours, colleagues, etc. and journal entries frequently contain anecdotes with sufficient detail to isolate these relationships, revealing how people are connected and thus de-anonymising participants.<br><br>We feel as a collective group that the significant redactions required to make the data shareable without 'outing' participants in many cases would render it overly thin for others to make replicative use for their own (re-)analysis. This is both a challenge of the method (PEER) we used in the	A separate email will be sent to the Editors seeking an exception to the data availability.<br><br>Ethics section, lines 261-265<br><br>Supplements 1 and 4

	project which purposefully selects known social contacts and the nature of sensitive research which underpins much of their journal entry content.<br><br>Our informed consent processes for both the PEER participants and this study's participants (which are necessarily intertwined) both stipulate:<br><br>• "…[personal] information will never be shared in a way that could identify who you are"<br>• "The contents of your sessions will be used only for analysis and for illustration in academic conference presentations and lectures. No other use will be made of them without your written permission, and no one outside the project will be allowed access to the original notes"<br>• "No one will be able to identify study participants unless they would like to have their [work] credited publicly"<br><br>We have taken steps to demonstrate transparency and authenticity of our work short of providing the data set as an open resource. These steps include:	

	1. Providing illustrative quotations throughout<br>2. Informing readers that S4 provides the full codebook<br>3. Adding Supplement (S1) showing the evolution of early coding to the final framework for 1 selected Global Theme (Learning). This Supplement shows a subset of Learning theme data. It provides more illustrative quotations that do not have sensitive anecdotes about participants and is thus low risk to authors and participants for re-identification.<br>4. We explain the rationale for data sharing in the Ethics section	
We have noticed that you have uploaded Supporting Information files, but you have not included a list of legends. Please add a full list of legends for your Supporting Information files after the references list.	This has now been amended and can be found following the References as per Editor request.	Following References (lines 841-852)
**Have the authors made all data underlying the findings in their manuscript fully available (please refer to the Data Availability Statement at the start of the manuscript PDF file)?**<br><br>The PLOS Data policy requires authors to make all data underlying the findings described in their manuscript fully available without restriction, with rare exception. The	Please refer to response above for information re: data availability	A separate email will be sent to the Editors seeking an exception to the data availability.<br><br>Ethics section, lines 261-265

data should be provided as part of the manuscript or its supporting information, or deposited to a public repository. For example, in addition to summary statistics, the data points behind means, medians and variance measures should be available. If there are restrictions on publicly sharing data—e.g. participant privacy or use of data from a third party—those must be specified.		Supplements 1 and 4
Results		
Your manuscript is missing the following sections: Results. Please ensure these are present, and in the correct order, and that any references to subheadings in your main text are correct. An outline of the required sections can be consulted in our submission guidelines here:<br><br>https://journals.plos.org/globalpublichealth/s/submission-guidelines#loc-parts-of-a-submission	We originally chose to present our results as a combined 'Findings and Discussion' section; for clarity, we have now renamed this "Results and Discussion"	Results and Discussion section (line 268)

Reviewer comment	Author reply	Location of changes
**Reviewer 1 comments**		
General comments		
This manuscript reports learning from a case study in which community-based researchers contributed significantly to particularly sensitive mental health research conducted in Sri Lanka. Employing community-based researchers is a growing practice and has potentially immense benefits for research outcomes and impact, the community-based researchers themselves. The strategy also comes with risks, which need to be mitigated and managed strategically from the outset. This manuscript presents a rigorous and thoughtful leap towards doing so and is likely to be hugely appreciated by research teams seeking to employ these methods safely and ethically, in LMIC and other settings. I have only three suggestions for the authors to consider.	Thank you to the reviewer for your positive feedback on the potential usefulness of this manuscript. We have addressed your feedback for each specific item below and hope you will find it satisfactory. Your inputs have motivated us to make clear improvements to the work and we are very grateful for Reviewer 1 having taken the time to show genuine interest in this work.	N/A
First, some funders and publishers require datasets to be open access. Although there can be clear ethical and other reasons to argue against this in individual cases, it is a pity if a solution to this cannot be found in similar circumstances as the current case study presents. I encourage the authors	We recognise the value of providing raw data where possible and considered carefully where to seek publication understanding the PLOS GPH priority for data availability.	A separate email will be sent to the Editors seeking an exception to the data availability.<br><br>Ethics section, lines 261-265

to consider including in their recommendations ways to allow similar data sets to be open access, if at all possible, and to demonstrate one solution for the data set on which the manuscript is based.	On November 21, 2021, we inquired with a member of the PLOS GPH editorial board (via email) prior to submission to understand whether an exception in this case would be considered. We explained that we felt this was the right forum for this specific piece, but also recognised the challenge of not having a fully open dataset.<br><br>Our authors are very closely linked with participants, including family, friends, neighbours, colleagues, etc. and journal entries frequently contain anecdotes with sufficient detail to isolate these relationships, revealing how people are connected and thus de-anonymising participants.<br><br>We feel as a collective group that the significant redactions required to make the data shareable without 'outing' participants in many cases would render it overly thin for others to make replicative use for their own (re-)analysis. This is both a challenge of the method (PEER) we used in the project which purposefully selects known social contacts and the nature of sensitive research which underpins much of their journal entry content.	Supplements 1 and 4

	Our informed consent processes for both the PEER participants and this study's participants (which are necessarily intertwined) both stipulate:<br><br>• "…[personal] information will never be shared in a way that could identify who you are"<br>• "The contents of your sessions will be used only for analysis and for illustration in academic conference presentations and lectures. No other use will be made of them without your written permission, and no one outside the project will be allowed access to the original notes"<br>• "No one will be able to identify study participants unless they would like to have their [work] credited publicly"<br><br>We have taken steps to demonstrate transparency and authenticity of our work short of providing the data set as an open resource. These steps include:<br>5. Providing illustrative quotations throughout<br>6. Informing readers that S4 provides the codebook<br>7. We added a Supplement (S1) showing the evolution of early coding to the final framework for 1 selected Global Theme (Learning). This Supplement shows a subset of Learning theme data. It provides more illustrative quotations that	

	do not have sensitive anecdotes about participants and is thus low risk to authors and participants for re-identification.<br>8. We explain the rationale for data sharing in the Ethics section	
Methods		
Table 1 (Community-131 based researcher characteristics) might benefit from an additional column providing whole-country demographic information on the relevant dimensions for comparison purposes.	We have now included national proportions based on the latest census data (2012) for available characteristics in the text, however these show total and not youth-specific characteristics. Unfortunately, accurate and contemporary data disaggregated for youth are not available through national datasets. For this reason, we chose not to add a separate column to the table itself, however we mark national proportions in the table where available and highlight these issues in the text.	Study sample, team positionalities and reflexivity, lines 140-155<br><br>Table 1, lines 157
Results		
Third, the analysis is rigorously conducted and evidenced and provides an excellent basis for the recommendations articulated. However, it is presented as a list. My experience is that usually themes/sub-theme analysis can be taken to the next level of analysis in the form of a model. At this next level, inter-relationships between themes and sub-themes are identified, possibly with feedback loops	We are very grateful for Reviewer 1's support for this particular issue. After reviewing their recommendations, and reconvening the authors, we felt that the most practical extension of our recommendations would be to share at what time point in a research or global health project teams could (and probably should) start thinking about key issues and ensuring resources are allotted for.	Results and Discussion, newly added text (line 638-659) and Figure 1 caption (lines 662)<br><br>Figure 1 separately uploaded

and posited intervention points. If the authors would like me to share an example I am happy to do so privately. I am thinking a particular study (currently in re-review) in which I was encourage by my collaborators to take a list of themes to the next level of integration which allow us to have a much clearer view of the processes involved in implementing change.	We considered a theory of change specific to our own project, however given the niche nature of the PEER method, we felt a broader global health project framing would speak to a wider audience.<br><br>We have therefore conceived this as an 'Opportunity timeline'. Rather than a theory of change in a traditional sense, junior colleagues proposed this can be a visual way for team members quickly plot when in a typical project they may be able to start having more say – and also when to identify that stakeholders working on projects before them have a responsibility to establish better conditions.<br><br>We welcome feedback as to the utility of this Figure.	

Reviewer comment	Author reply	Location of changes
**Reviewer 2 comments**		
General comments		
Overall commend the concept of this study – important to examine and consider this topic and interesting methods and reflexivity – thank you	Our thanks to Reviewer 2 for their support towards improving this manuscript and their numerous inciteful requests. We feel your inputs have significantly improved the paper.<br><br>In particular, we would like to appreciate their curiosity for more – not less – information. We had omitted certain details in the original manuscript on account of word count, but are grateful someone has taken a sincere interest in how we worked through this study and as a team.<br><br>We have attended to each of your comments below and hope our responses and changes are satisfactory.	N/A
Abstract		
Abstract - English needs further editing – some of the sentences had to be read several times to understand – an edit to get a brisk and clear meaning recommended – eg. This sentence in abstract "Participatory methods, which rely heavily on community-based data collectors, are growing in popularity to deliver much-needed evidence on	We have broken up several longer sentences to make the content easier to digest.	Abstract, lines 3-29

violence and mental health in low- and middle-income countries facing the greatest burden, but fewest resources to respond".		
Introduction		
Introduction – Sets the scene – however the sequence of paragraphs could be reviewed e.g. in first paragraph the objective of study is stated – (lines 42 -44) and this is then replicated with expansion at end of Introduction section - generally feel that it could be more concise with less repetition.	We have made amendments to the Introduction to reduce repetition between the initial signalling of the rationale for the paper and the specific introduction of what we did. Our wider group was keen to preserve mention of this paper's co-production early on in the manuscript as so few studies have been able to do this to date.	Introduction, lines 47-49, 84-98
Introduction - While in 36 – 38 the authors outline growing popularity of participatory methods – they have not engaged in any depth with the reasons for its' importance, and have not engaged with some key discussions around decolonisation in global health/ the power relations in community coproduction of knowledge eg Abimbola S. The uses of knowledge in global health. BMJ Specialist Journals; 2021. Or eg 2 Abimbola S, Pai M. Will global health survive its decolonisation? **Lancet. 2020;396(10263):1627-8.**	Many thanks for raising this. We have incorporated 2 additional references in the Introduction and Conclusion section of the paper now as per reviewer feedback.<br><br>Reviewer 2 may find our experience of interest:<br><br>We held numerous discussions amongst our team throughout the project (and continue to do so now) about matters of decolonisation and shared others' writings on this issue to discuss and debate. We acknowledge that different groups prefer different strategies for thinking about this issue.	Introduction, lines 44-47<br><br>Discussion and Conclusion, line 624-628

	In specific discussions around writing this paper, and for our project language more generally, there was vocal and clear preference not to use the term "decolonisation" or to frame the paper as an exercise in decolonising literature. The term itself was viewed by some partly as an HIC and somewhat academic/detached export and was experienced by other team members as rhetoric which did not mean much in practical terms. As a team, we were conscious not just of power imbalances borne out of legacy HIC-LMIC hierarchies, but locally produced power structures (not tied to colonial histories) that were often perceived much closer to CBRs' lives impacting their (in)ability to participate in certain spaces, including careers like global health and development. Certain professional spaces continue to be shaped by caste, class and ethnicity structures which may be missed by a decolonising discourse.<br><br>In short, we held explicit conversations in which team members requested to focus on concrete behaviours and choices that democratised research in this setting over the decolonisation narrative.<br><br>We imagine there is indeed a paper in there just on our internal discussions about this very thing! In the	

	meantime, we have aimed to strengthen mention of power and small ‘p’ politics in global health at higher levels and within our particular team and project.	
Methods		
Methods – given the challenges of including CBRs in research question design – it would be useful to describe how or whether CBRs were involved in writing the grant for this research – and if they were – how they participated – and if they weren’t, how the grant proposal gave space for new question development. Please elaborate as the inception of this study is relevant to the power relations and peer researcher proposal and to understand the primary investigator/ core team roles vs the CBR roles.	In our original manuscript, we selected to omit in depth details of the broader research grant on account of word count. However, we are pleased to re-introduce clarity around this process, which was a silver lining to our otherwise very challenging circumstances in COVID. We draw the reader’s attention to our choice to explore participatory methods following original grant plans being disrupted, which opened up the research project in a new way and allowed young CBRs to have early say in specific topics and other core decisions.<br><br>It is worth noting that 2 of the 4 core team members are also minority youth who had also not previously had a chance to ideate research projects from the ground up on previous occasions. This was an unexpected and beneficial experience for the entire team to have more control over what we ultimately explored.	Study Setting and Scholarly Context, lines 114-136
6. Methods - Stance and reflexivity – core team/ CBR – while this paper includes many aspects of reflexivity, it would be useful to understand the	We have now expanded on our sub-section Study sample, team positionalities and reflexivity.	Methods: Study sample, team positionalities and reflexivity, lines 169-187

privilege/ background of authors and how the core team vs CBR relationships were managed to minimise hierarchical power relations – please add this to the Methods section	Herein we address the conscious efforts we made, and what we did not do, to support a flatter team dynamic.<br><br>We address the specific issue of technology inequalities in a separate location in the Results and Discussion, in response to your comment 2 rows below, but acknowledge he this does relate to power to participate.	 <br><br><br>Results and Discussion: Experiences of (support from) the core team, lines 589-600
Methods - Recruitment of CBR – given the impressive representativeness and diversity – it would be good to understand further detail on how the CBR were recruited.	A more detailed explanation of how we found CBRs has been added to the Study setting and scholarly context sub-section within the methods.	Methods: Study Setting and Scholarly Context, lines 119-122
Discussion/Conclusion		
Findings/ discussion – the four themes are relevant and informative and discussion of each theme engages well with relevant literature. It would be useful to include further discussion of the ways that technology/ access to technology influenced the research process and contributions e.g. were there ways that more remote CBRs could not use their video's on during ZOOM or other meetings which reduced their sense of participation? Some further discussion of technology and how this can democratise or exclude in particular would be appreciated.	We now address technology inequalities explicitly in the Discussion section with new text:<br><br>"In particular, technology like phones, cameras and laptops, and associated running costs were critical resources for remote participation. All CBRs had access to basic smart phones and internet connection and were furnished with data in advance so as not to disadvantage any individual's access to internet throughout the study. Other inequalities in technology access were addressed through offers to provide devices to CBRs according to their needs to	Results and Discussion: Experiences of (support from) the core team, lines 589-600

	enable fair participation. Some CBRs chose not to accept additional devices (e.g., laptops), preferring to work in other mediums, and the core team respected these decisions as it did not impact CBRs' ability to join discussions nor to deliver quality work. Other projects may require more uniform use of technologies."<br><br>We previously raised technological challenges that affected CBRs feeling connected to participants during the parent study – largely due to weather disrupting tech rather than lack of access to technology in itself.	 <br><br><br>Identified challenges, lines 469-473
Table 2 is a good summary – however I wonder if there is a role for any 'rest stops' in the study process where team members take time for discussion about the coproduction process – any points to elicit explicit reflexivity about positions/ power relations and how these influence interactions within the team as well as CBR with study participants. See the Schaaf paper you have cited and perhaps hold up your process to consider whether it has fully engaged with all the ways power relations can influence coproduction and participation.	We have now expanded our discussion on navigating power dynamics in the team in our Methods section, specifically under "Study sample, team positionalities and reflexivity". Herein we describe our actions to open up participation and welcome continuous feedback.<br><br>We clarify the use of debriefs as 'curiosity sessions' in which CBRs were invited – and vocal! – to feed back on the process of doing research, not just on the content being generated through it. We very	Methods: Study sample, team positionalities and reflexivity, lines 169-187

	much like this language of 'rest stops' and will consider how to incorporate this going forward.	
Discussion – can you please elaborate on how CBRs participated in analysis/ paper writing given English medium and academic content – e.g. thematic analysis/ discussion	We have chosen to address this in the Methods section itself, bridging the issue of data analysis and co-production of the paper itself in one subsection.<br><br>We appreciate the interest in this particular issue as it was the first time any of us has co-written across the 3 languages before and was a huge undertaking, and something we are proud we pushed ourselves to do. This is especially the case doing it remotely during the pandemic and now amidst the country's worst humanitarian and economic crisis since its independence. We have had a complex struggle to connect 25 of us across completely different power cut schedules and competing pressures of living amidst the crisis. We are certainly hoping to be able to celebrate everyone's achievement when we can finally get together again.<br><br>A subset of CBRs are interested in translating the full recommendations checklist (i.e., Table 2) and an executive summary of the paper into Sinhala and Tamil for wider use around Sri Lanka as local	Methods, Data analysis and co-production of the paper, lines 238-248

	language research guidance is lacking. However, we chose to wait to do this until a final manuscript is ready. We would welcome the journal's consideration of a Supplement of these items to be added later to the article should it be accepted for publication. We will pursue dissemination of these items internally also through our respective networks.	
